# Carpenter's Principles of Human Physiology

**Published:** 1853-01

**Authors:** 


***Carpenter's Principles of Human Physiology.*** Philadelphia, Blanchard & Lea, 1853. (5th Am. Ed.)

THIS is edited from the fifth English edition, and no pains seem to have been spared by the American publishers to make it worthy the reputation of the distinguished author.

Of the character of the book, it can hardly be necessary for us to speak to our intelligent readers. Carpenter's contributions to physiology are in themselves valuable, and the indefatigable industry with which he collects and classifies the discoveries of others, and the cautious yet candid spirit which characterizes his own deductions, give to his writings the authority which the partizan of a particular school can never attain. In the edition before us, he has labored most faithfully and zealously at the perfection of his book and has made several important alterations, while he has presented the very latest facts in the science, sifted and freed from the falsehood that might adhere to them. He has changed the order of his chapters, we think for the better. He has added an entirely new chapter on physiological chemistry, taken chiefly from Lehmann's new work. This is an important addition, though, in our opinion, he pronounces too positively in favor of Liebig in the Protein controversy. We cannot think that Müilder's experiments have been so entirely invalidated by Liebig's researches, and his deductions so completely refuted, as the first part of this chapter would lead the reader to suppose. Claude Bernard's recent results find a suitable place, and Kölliker's late investigations are also contained in this edition. Altogether, it fully sustains the well-earned reputation of its accomplished author.

